# Supplementary material for: Virological characterization of a new isolated strain of Andes virus involved in the recent person-to-person transmission outbreak reported in Argentina
Source: PLoS Negl Trop Dis. 2025 Jun 16;19(6):e0013205. doi: 10.1371/journal.pntd.0013205 (PMC12201636; doi:10.1371/journal.pntd.0013205)
Supplement: Attachment — Submitted filename: Reviewers respons.docx [file pntd.0013205.s004.docx]

# **Reviewer #1**

Thank you very much for your suggestions and for taking the time to make detailed corrections. We carefully reviewed your comments and agree with all of them. In our opinion, this work is much clearer now. We believe the concerns expressed in the minor revision have been clarified in this new presentation. Below, we provide a detailed response to all of your comments.

*Methods*

**1. Reference 22 is very important, as it is listed as the reference for the sequencing approach, but the reference is incorrect—it is a bioRxiv but I believe is now published in PLOS NTD.**

We have updated the reference to include the latest version published on PLOS NTD (reference 22)

**2. Line 249, what MOI was used?**

This value was only shown in the X-axis of Figure 3, but we added it text (line 258 in the new version). The MOI was 1 in both cell lines, A549 and Vero E6.

**3. Line 125-142, what size flasks were used for infection at each step?**

All steps were performed using T25 flasks, that information was added to the manuscript in line 132.

**4. Where was the anti-ANDV serum obtained and how was it made?**

Thank you for this observation. The anti-ANDV serum was made in rabbits by immunization with recombinant nucleoprotein. The procedure was explained in Padula PJ et al 2000 (reference N°20). This reference was added after the sentence (line 155 in the new version).

*Results*

**1. Figure 1 legend states there are panels A-F, but there is only A and B on the figure itself.**

Thanks for the observation, this was a mistake during the uploading process. We now uploaded the correct file.

**2. Figure 2, why are there no time points for ARG after day 12 like Epuyen?**

This was because the strain ARG was used as a control only. Our objective was to characterize Epuyen. Furthermore, after this well characterized strain reaches its highest infective titer (Day 5 after infection in this figure), and tends to decrease, irreversibly.

**3. There is no text in the Figure 2 and Figure 3 figure legends.**

Thank you very much for pointing out this mistake, we added legend for both figures

**4. FFU data is needed for Figure 3.**

Infective particles were not quantified for this experiment. Our intention was only compare the kinetics of replication of both strains in two different cell lines.

**5. Table 1 should describe at what timepoints post-infection the viral RNA was tested in lung and blood and when the IgG titers were tested.**

As it is explained in the text (lines 182-186 in the new version) all the animals were sacrificed at day 30 pi and were sampled. The exceptions were those that died during the night (only two individuals). In order to minimized the handling of infected hamster, each one was sampled only once. We added the information at the footnote of the table as follows:

At day 30 post infection blood samples were collected from all the hamsters and then were sacrificed and necropsied. The exceptions were MR-1 and MR-2 which were found dead at day 16 and 22 respectively, then lung samples were obtained post mortem

**6. Please show negative controls for ANDV staining in Figure 1.**

Thanks for the observation, we added the figure corresponding to the Control (mock infected cells)

*Conclusions*

**Lines 324-326, 377, and 282, can it be stated that the isolated virus is a wild-type strain and one that doesn’t need adaptation for in vitro growth? It has a mutation in the non-coding region of S and a coding mutation in L. As a comparison, the vaccine TC-83 strain of Venezuelan equine encephalitis virus is attenuated due to only 2 mutations compared to the wild-type strain—one in the 5’ UTR and an amino acid change in the glycoprotein. While the number of changes is small in this manuscript after isolation—and impressively so, given the difficulty of isolating hantaviruses—some nuance should be used to discuss “wild-type” and “adaptation.”**

Your comment is really interesting and we understand your point. As little is known about structural and functional properties of the RdRp, any prediction about the impact of this aa change is speculative, even more so the C-terminal end of the protein is poorly described. So, we decided to change the sentences you have pointed out as follows:

We replaced “ARG-Epuyén grew in cell culture without need of adaptation” with “The isolated ARG-Epuyén strain accumulated only two mutations” (line 282 in the new version).

This part of the sentence “indicating that it can be considered a wild-type strain” was deleted from line 325-326 from the original version (line 328 in the new version)

Line 377 from the original text: “Therefore, it can be considered an ANDV wild-type strain, representing a critical step toward developing MCMs against this emerging pathogen.“ was changed by this one: “Future studies will be necessary to determine if these two changes are attenuation determinants for the hamster model, or whether they lack pathogenic impact and therefore this strain could be considered an ANDV wild-type strain. If so, it would represent a critical tool toward developing MCMs against this emerging pathogen.” (lines 379-383 of the new version)

*Editorial and Data Presentation Modifications?*

**1. The manuscript should be reviewed for English grammar and style. Below are a few changes that should be made:
Line 77, please change “After infection, the signs and symptoms of the disease can manifest after a long period of up to 40 days” to “After human infection, the signs and symptoms of the disease can manifest after a long incubation period of up to 40 days”**
**Line 89, change “could be” to “was”
Line 99, change “since” to “and”**

Changes were done.

# **Reviewer #2**

We thank you for taking the time to review our manuscript and for your thoughtful suggestions and comments. Your observations were very helpful in improving the clarity and quality of our work. We especially appreciate the opportunity your feedback gave us to further reflect on some key aspects and to slightly expand the discussion where appropriate. Below, we respond point by point to each of your comments.

*Methods*

**The objectives and design of the study are well-defined.
Regarding concerns about regulatory aspects, the manuscript states that the experiments in the animal model were conducted in a safety level 3 facility; BSL4 is typically required.**

All the experiments involving animals were performed in an animal BSL-3 facility (ABSL-3), using BSL-4 practices. It is noteworthy, that this facility is an ABSL-3 *plus* facility because it is equipped with class III biological safety cabinets, individual ventilated caging system, effluent decontamination system, and exit showers. Among special practices, only trained personnel with more than 10-year experience were involved in the manipulation and sampling of infected hamsters. Animal handling was minimized to what was strictly necessary. Since no such facilities existed in South America and due to the need to characterize a new strain of this virus, which is endemic to Argentina and Chile, it was decided to use the ABSL-3 plus facility under the strict supervision of biosafety experts. This shortcoming led to the decision of the authorities of ANLIS and Ministry of Health to build a BSL-4 laboratory, which is about to open. Future experiments involving ANDV infections in hamsters will be perform in this new BSL-4 facility.

*Results*

**The analysis and results are written in an orderly and clear manner, with their respective subheadings.
The tables and figures are highly relevant, well-prepared, and easy to understand.
Table 1 establishes that there are 18 rodents in cages 1 to 7, and the text establishes that there are 17 in 7 cages.**

Thank you for this observation. Effectively there were 18 rodents as indicated in table 1. We corrected the text Line 174 in the new version.

*Conclusion*

**I believe the discussion is appropriate and well-informed; I would suggest focusing the discussion more on transmission (person-to-person) than on pathogenicity.
It would be interesting to know the authors' opinion on the observation that a significant viral load is detected in some rodents, yet no antibody response is observed.**

The hamsters with high viral load but no antibody response at day 30 after experiment beginning are those which were infected by the index hamsters (index hamsters: those that were intramuscular inoculated, one per cage). Therefore, the viremic curve is growing up while the humoral immune response is not detectable yet.

Particularly, for the two hamster that succumbed, MR-1 and MR-2, and that developed low IgG titers, it is worth noting that human HPS cases with low IgG antibodies titers were associated to fatal outcome.

*Summary and general comments*

**Isolating the Andes hantavirus from a human is a significant achievement, even more so if this isolation comes from a patient involved in a person-to-person outbreak of the magnitude of the one that occurred in southern Argentina. It is no exaggeration to say that this isolation will contribute to a better understanding of the epidemiology of the infection and the development of therapeutic and control tools. The paper is well-written, with well-founded statements, and the study design and methodology are appropriate. The characterization of the infection dynamics in cell lines, the genetic studies with sequencing, and the evaluation of virulence and transmissibility in the Syrian hamster animal model were very well executed and provide relevant information.
The only general criticism that can be raised about this work is that the title may suggest that the objective of the study is to try to elucidate whether the virus involved in a person-to-person outbreak has some special characteristic that explains why this virus acquires a capacity that is unusual in hantaviruses. I would suggest that the discussion analyze whether some of the substitution changes in the M segment could have made the virus capable of transmitting to humans, or speculate on what circumstances or virological properties could have led to this epidemiological event.**

**It is very interesting that the viruses tested are transmissible between hamsters, and intriguing that the virus isolated from a human does not cause disease in this model. Both results (transmissibility and lack of disease development with human viruses) confirm previous findings, adequately described in the paper.**

Regarding your suggestion to analyze changes associated to PTP in the discussion, we added a paragraph regarding this topic (Lines 348-351, new version). However, as we have few complete genomes of strains well characterized (implicated in PTP or not) all the conclusion we could made would be only speculative. For example, ANDV/ARG was isolated from a rodent; we don’t know whether it is pathogenic in humans or even if it could be transmitted PTP. Then, we need to obtain new well characterized strains and/or new complete viral genomes associated to PTP HPS cases and from zoonotic transmission as well.

# **Reviewer #3**

We would like to express our sincere gratitude for the time you have dedicated to reviewing our manuscript and for your valuable comments and suggestions. We appreciate your analysis, which has significantly contributed to improving the quality and clarity of our work. Below, we provide detailed responses to each comment, including the changes made.

*Methods*

**The objective of the study is clear and well design to address the Virological characterization of new Andes virus strain and to support the conclusions. All experiments are presented, not in detail, but well referenced. There are no concerns about ethical requirements, but I ask for the number of the protocals and permissions to be included in the text.**

The document was approved by the “Comité de Bioética Comodoro Rivadavia” (Secretary of Health from Chubut province, Argentina) under the number 4/2024.

*Results*

**All results match the analysis plan, and are clearly presented. With the exception of figure 1 all images and tables are of sufficient quality.**

Thanks for the recommendation, we improved the quality of the Figure 1

*Summary and general comments*

**Reviewer #3: Line 21 Andes virus (Orthohantavirus andesense)**

As the taxonomic status was clarified in the introduction section, we prefer leaving the abstract as it was. This is because the numerous changes that were introduced by the ICTV has generated confusion in people. In particular, in this case we don´t want that “Andes virus” be confused with the taxonomic rank, “*Orthohantavirus andesense*”

**Line 47 but none of them were**

Done.

**Line 49 and has an unclear history**

Done.

**Line 58 please refer to the updated taxonomy of hantaviruses (Bunyaviricetes: Elliovirales: Hantaviridae: Mammantavirinae)**

Done.

**Line 112 use PTP that has already been cited in the text**

Done.

**Line 117 use HPS that has already been cited in the text**

Done.

**Line 165 how many days? Please specify.**

Done.

**Line 171 … transmission, Syrian Golden Hamsters (Mesocricetus auratus)**

Done.

**Line 197 TRIzol**

Done.

**Line 218/19 – provide the protocols number for the ethics permissions.**

Done.

**Line 223 - how many days after the probable exposure?**

The patient was probably exposed around 16 to 18 days before the beginning of symptoms. This is uncertain because it is not known the exact day that an ill person is contagious.

**Table 2 – please include GenBank accession number.**

Done

**Line 299/300 – use MCMs that have already been cited in the text**

Done

**Line 312 – Hantaviridae needs to be in italic**

Done

**Line 318 2^nd^**

We understood that you mean that the correct form is November 2 and changed it in the manuscript.

**Throughout the text the terms “in vitro” and “in vivo” need to be in italic**

Done
